# The Influence of Steeping Water Change during Malting on the Multi-Toxin Content in Malt

**DOI:** 10.3390/foods8100478

**Published:** 2019-10-11

**Authors:** Kristina Habschied, Rudolf Krska, Michael Sulyok, Jasmina Lukinac, Marko Jukić, Bojan Šarkanj, Vinko Krstanović, Krešimir Mastanjević

**Affiliations:** 1 Josip Juraj Strossmayer, Faculty of Food Technology, University of Osijek, Osijek, F. Kuhača 20, 31000 Osijek, Croatia; ptfosptfos2@gmail.com (J.L.); ptfosptfos@gmail.com (M.J.); vkrstano@ptfos.hr (V.K.); kmastanj@gmail.com (K.M.); 2 Institute of Bioanalytics and Agro-Metabolomics, Department for Agrobiotechnology (IFA-Tulln), University of Natural Resources and Life Sciences, Vienna (BOKU), Konrad–Lorenz-Straße 20, 3430 Tulln, Austria; rudolf.krska@boku.ac.at (R.K.); michael.sulyok@boku.ac.at (M.S.); 3 Institute for Global Food Security, School of Biological Sciences, Queens University Belfast, University Road, Belfast BT7 1NN, Northern Ireland, UK; 4 Department of Food Technology, University North, University center Koprivnica, Trg dr. Žarka Dolinara 1, 48000 Koprivnica, Croatia; bsarkanj@unin.hr

**Keywords:** barley malt, *Fusarium graminearum*, malting, multi-toxins, steeping regime

## Abstract

The aim of this study was to assess the impact of steeping water change and *Fusarium graminearum* contamination level on different multi-toxin types and concentrations in barley malt. Malt samples were subjected to two micromalting regimes—steeping water change and the other with no steeping water change. Malt was contaminated with different *F. graminearum* contamination levels (0%, 10%, and 20%). The results indicate that malt with higher *F. graminearum* contamination levels ensured higher concentrations of toxins. Higher fungal metabolite concentrations were determined in samples exposed to freshly-changed steeping water, especially zearalenone and its derivates whose values were three to four times higher than in samples with no water change. Zearalenone-4-sulfate showed four (in 10% contamination) and even thirty times (in 20% contamination) higher concentrations than in samples with no water change. Water change during malting resulted in higher levels of multi-toxins in the final product.

## 1. Introduction

Barley grains are nutritionally rich and they are; therefore, suitable for microbial growth and proliferation, especially during the malting process. Fungi from genus *Fusarium* are naturally present on the grain and may cause infections and economic losses for maltsters and brewers, especially during rainy years [1]. Fungi can additionally spread during transport and in storage facilities if the grains are not stored properly (appropriate temperature, aeration and air humidity). In order to avoid fungal proliferation, the initial grain moisture should not go over 13%. The malting process ensures extremely favorable conditions (temperature, aeration, and humidity) for fungal growth and mycotoxin production [2,3]. *Fusarium* fungi can cause great economic losses and that is why maltsters are obligated to refuse a batch that shows any symptoms of *Fusarium* infection. *Fusarium* infection results with recognizable reddish grains, as described by the European Brewery Convention (EBC) or Mitteleuropäische Brautechnische Analysenkommission—MEBAK^®^ [4,5]. *Fusarium graminearum* is one of the most widely spread fungi in Europe and its varieties can produce toxins when fungus undergoes unfavorable and stressful conditions [6]. To this day, several hundred mycotoxins have been described [7]. Mycotoxins from cereals transfer into malt and to the final product, beer, where they can be detrimental to human health [8,9,10,11,12]. In the malt–beer chain, most studies consider trichothecenes as the most relevant mycotoxins due to their properties of being water-soluble and resilient to high temperatures at lower pH values (such as alcoholic medium—beer) [13,14]. Other important mycotoxins in the malt–beer chain are aflatoxins, fumonisins, ochratoxin A, and zearalenone (ZEN). One of the most studied *trichothecenes* is deoxynivalenol (DON, vomitoxin) that has been specified as one of the most important indicators of quality and safety of malting barley. As a secondary metabolite of *Fusarium* species, ZEN is among the most frequently detected mycotoxins in cereals. During malting and brewing processes mycotoxin concentrations vary, because synthesizing or releasing from conjugated/modified forms occurs throughout the different production stages. Modified mycotoxins, formed by different metabolic reactions via plant defense system or microbial metabolism (glycosylation, acetylation, etc.), usually co-occur with the basic form of mycotoxin (DON and DON-3-GLC) [15]. Rychlik et al. [16] defined modified mycotoxins as conjugation products synthesized via the detoxification mechanisms of living organisms. In that sense, the fermentation process during brewing purports the use of brewer’s yeast that can metabolize mycotoxins [17,18], which adopt a less toxic form. However, some microorganisms and unit operations can reverse the biotransformation and contribute to the toxicity of modified forms [15].

Although mycotoxins do not disturb the brewing process, in a sense of hindering or stopping the fermentation, except in very high concentrations (>10 mg/L of DON) [10,14], they can have a great influence on the human health. According to several papers, commercial beers can contain various amounts of different mycotoxins in very low concentrations (<1 µg/L) [19,20,21,22,23,24]. DON, nivalenol (NIV), T-2, HT-2, DAS, ZEN, aflatoxins, ochratoxin A, and fumonisins were determined in the analyzed beer samples in concentrations at, or slightly above, the tolerable daily intake (TDI) [24]. Known mycotoxins are not the only toxins that can be found in malt and beer. The emerging multi-toxins are getting more and more attention since they can cause health problems to consumers [25].

This research was designed led by the hypothesis that steeping water retains water-soluble myco/multi-toxins as our previous work confirmed [11]. For that matter, different degrees of *Fusarium* infection and steeping regimes were compared with their influence on the multi-toxin concentrations in malt.

## 2. Materials and Methods

### 2.1. Sampling

Barley (*Hordeum vulgare*) variety Pivarac, used in this research, was obtained from the Agricultural Institute in Osijek, grown at location Osijek (45°27′ N, 18°48′ E) and harvested in June 2018. Grain samples (5 kg) were collected as untreated and conditioned grain, scaled and packed into in double-walled paper bags (1 kg). Until micromalting, the material was stored in sterile dry containers for two months in a dry and cool place (18–20 °C) to overcome post-harvest grain dormancy. Barley samples were infected with *Fusarium graminearum* (CBS 110250, Centraalbureau voor Schimmelcultures, Utrecht, Netherlands) during steeping and germination phases. In order to obtain the infected malt, the inoculation of barley was carried out by adding mycelial discs onto barley during the germination phase on the first day. *F. graminearum* mycelium was prepared as described by Habschied, Šarkanj, Klapec and Krstanović [26]. In short, PDA (potato dextrose agar) was used for fungal growth with incubation temperature set to 14 °C in order to reduce the shock after inoculation of malt, since the malting temperature was 14 °C. Five mm diameter discs were cut from the margin of the *Fusarium* colony. All incubations and analysis were performed in parallel, and all results were shown as average values.

### 2.2. Micromalting Procedure

Micromalting was performed as described by Mastanjević et al. [10] and according to MEBAK^®^ [4]. Before the micromalting took place, in order to reduce the existing microflora originating from the field and storage facility, barley grains were washed with 3% sodium hypochlorite (*v*/*v*) for 5 min and rinsed with sterile water [27] Five hundred grams of barley were soaked in 500 mL of tap water, according to the procedure described in MEBAK^®^ [4], and shown in Table 1. In total, 4 batches (2 infected and 2 healthy) were malted, resulting in cca 2 kg of malt (1 kg of infected and 1 kg of control malt).

Two steeping models were applied. In the first batch of the infected sample, steeping water was regularly replaced after every steeping phase (AI), and in the second batch the steeping water was not changed during malting (BI). Identical procedures (AH and BH) were also applied for the healthy (control) barley during malting. The added water was the same temperature as the water in the tub before the replacement (14 °C). The kilning of green malt was also performed according to the MEBAK^®^ protocol. After drying, malt was transferred into paper bags and kept at a room temperature for three weeks for moisture equilibration. Microbiological analysis of control (healthy) barley showed 0% contamination with *Fusarium graminearum*, and after the malting procedure, the obtained malt (infected and healthy) was also subjected to microbiological analysis. A 100% contamination was established for the infected batch and 0% contamination for the control (healthy) one. In order to acquire different contamination levels (0%, 10%, and 20%) the infected malt was mixed with the healthy malt sample (batch AI with AH and batches BI and BH) which was validated by using the microbiological method described below.

### 2.3. Microbiological Analysis

The actual *F. graminearum* contamination degree was determined in barley and malt samples according to the method described in MEBAK^®^ [4]. This procedure was conducted in triplicate, mean values were taken as a result and shown in Table 2.

### 2.4. Multi-Toxin Analysis

The multi-toxin screening was performed as described by Malachová et al. [28] in the Center for Analytical Chemistry, Department for Agrobiotechnology (IFA-Tulln) at the University of Natural Resources and Life Sciences, Vienna, Austria. Five grams of the homogenized ground sample was extracted with the extraction solvent acetonitrile:water:acetic acid = 79:20:1 during 90 min by using a GFL 3017 rotary shaker (GFL, Burgwedel, Germany) at 180 rpm and room temperature. Following, extraction crude sample was precipitated and 500 μL of clear extract was diluted with dilution solvent (acetonitrile:water:acetic acid = 20:79:1). For the separation Agilent 1290 UHPLC system was used combined with Gemini^®^ C18 (150 × 4.6 mm i.d., 5 μm particle size) column, and C18 security guard cartridge, 4 × 3 mm i.d., while the Sciex 5500 qtrap^®^ system was used for detection and quantification. All system parameters were as described in Malachová et al. [28]. All samples were analyzed in triplicates.

### 2.5. Statistical Analysis

Experimental data were analyzed by the analysis of variance (ANOVA) and Fisher’s least significant difference (LSD), with significance defined at *p* < 0.05. Statistical analysis was carried out with Statistica 12.7 (StatSoft Inc., Tulsa, OK, USA).

## 3. Results and Discussion

The results of conducted research are presented in Table 2, Table 3, Table 4, Table 5 and Table 6. In order to obtain the wanted microbiological contamination, the infected malt was mixed with the healthy one and the final contamination level was verified according to the standard MEBAK^®^ [4] procedure. The results of microbiological analysis are presented in Table 2.

In several research articles published by Krstanović et al. [29,30] and Velić et al. [31] monitoring of *F. graminearum* and *F. culmorum* in Croatian barley and wheat varieties was conducted. In order to obtain statistically significant changes on fungal contamination, Krstanović et al. [30] examined the *F. graminearum* contamination of barley for three consecutive years (2001–2003) and reported a maximum average value of 19% of contamination. A previous pilot survey was conducted by Krstanović et al. in 2015 [13], where a relation between barley and malt contamination was followed. The results of their research showed that a 20% contaminated barley gives about three-fold lower results for malt contamination, resulting in 7% of contaminated malt. Encouraged by these results, it was concluded that a 20% contamination level for malt represents realistic agro-climatic conditions for Croatian barley. This is the reason why the maximum malt contamination level was kept at 20%.

The results of the determination of DON and its derivatives, so called modified mycotoxins, are shown in Table 3. The results obtained in this investigation indicate that steeping water regime significantly (*p* < 0.05) affected the production of (myco)toxins, in the respect that more (myco)toxins were synthesized in the batch where steeping water was regularly changed. For 20% contamination, DON production was higher by around 1.5 times in the batch A in comparison to batch B, where the steeping water was not changed. Similar situations occurred with DON derivates, DON-3-GLC (deoxynivalenol-3-glucoside), and 3-ADON (3-acetyldeoxynivalenol) in this case.

The concentrations of ZEN and its derivatives, ZEN-4-SULF (zearalenone-4-sulfate) in malt samples, are shown in Table 4. A similar trend was observed for ZEN and its derivatives as with DON and its derivatives. Namely, the batch with fresh water contained significantly (*p* < 0.05) higher levels of (myco)toxins in general, and especially ZEN-4-SULF, where four (10% contamination) to 30 (20% contamination) times more was found. ZEN also showed an increase of three to four times in samples where the water was changed during every steeping. Besides the fact that the levels of these mycotoxins were above the legislative regulation [32], such results are worrying in case of higher initial contamination. According to the BIOMIN World Mycotoxin Survey [33], the European crops, especially in the southern areas, are greatly affected by mycotoxins, with ZEN and DON being the most spread mycotoxins (61% for ZEN and 82% for DON). According to BIOMIN research, 14% of positive samples contained ZEN above the legal threshold, and 55% of DON positive samples exceeded the legal threshold. Such highly-contaminated cereals should be avoided for the malting to reduce the risk of heavy mycotoxin contamination of the final product—beer.

Other detected and quantified toxins are summarized in Table 5. Even though the concentrations are generally low, some of them may represent a serious threat to human health. Some of most concerning ones are *Alternaria* toxins, whose acute toxicity is rather low, but alternariol methyl ether has been proven to be mutagenic and genotoxic [34]. As seen from the Table 5, not only *Fusarium* toxins are detected in malt samples, but small amounts of different fungal and plant metabolites and toxins can also be found. Those toxins and/or metabolites are probably originating from the field contamination by natural mycobiota, or are part of the barley’s natural defense system (as the cyanogenic glucoside lotaustralin). This can be attributed to the remnant microorganisms on the grains before malting, despite the sodium hypochlorite rinse. Namely, since the barley grain has to retain its property to germinate, it cannot be sterilized using high temperatures and rinsing with sodium hypochlorite cannot ensure an entirely sterile sample. There is a possibility of remaining spores of *Alternaria* spp. after the sodium hypochlorite rinse, and the increase of *Alternaria* toxins with the increase of the infection rate. Additionally, the increased lotaustralin levels in the samples indicate that the activation of the plant’s natural defense system occurred (increasing the cyanogenic glucosides biosynthesis) when exposed to higher fungal contamination. This can be relevant when producing beer or whiskey, where they can be converted to cyanide by *Saccharomyces cerevisiae* β-glucosidase [35].

In general, opposite to our starting hypothesis that different myco-/multi-toxins concentrations would be lower in malt with water change, metabolite levels appeared to be higher in the case where the steeping water got changed during the steeping phase, at least for metabolites in Table 2 and Table 3. Given that the water is the only variable in this experiment, this can be attributed to the fresh batch of nutrients that came with tap water. One possible explanation could be that fresh tap water inflow increases the dissolved essential minerals and oxygen concentrations in the batch and; therefore, serves as a re-activator of the enzymes involved in the (myco)toxin biosynthesis [36]. The other theory would be that the fresh water acted unfavorably on fungal mycelium and stimulated mycotoxin production. According to the local water factory’s annual report on water quality intended for human consumption for 2017 [37], the amount of all metal ions was within the legal recommendation; except for arsenic of which the allowed concentration in drinking water is 10 µgL^−1^ and the determined concentration was 30 µgL^−1^. Since arsenic is a known oxidative stress activator, and arsenic levels in the used tap water were three times higher, this might have had an influence on mycotoxins production stimulation, as it is known that fungi synthesize mycotoxins when found in unfavorable and stressful conditions [6]. According to Cuero and Quellet [38], metal ions (Zn^2+^, Fe^2+^, Cu^2+^) have a stimulatory effect on zearalenone production in *Fusarium graminearum*. However, this has yet to be investigated. In some cases, 10% contamination resulted in higher mycotoxin concentrations than in 20% contamination. This is especially pronounced for fusarin C, rubellin D, rugulusovin, siccanol, and tenuazonic acid, and is probably a result of complex fungal metabolic pathways that can be influenced by many biotic and abiotic conditions. Perhaps high concentrations of these mycotoxins are in direct (or indirect) leverage with the production of some other mycotoxins. Co-occurrence of some mycotoxins is usual (as can be seen from Table 6), but the data on interactions between different mycotoxins, especially emerging ones, are lacking. Anyways, this is something that should not be neglected, but rather further investigated.

## 4. Conclusions

In order to investigate the effect of *F. graminearum* infection rate and mycotoxins diversity during malting, two malting regimes were applied, A) where steeping water was changed during malting and B) where steeping water was not changed during malting. The extensive multi-toxin analysis of malt samples showed an increase of monitored mycotoxins and other toxins in both cases depending on *F. graminearum* contamination degree. However, the results indicate that the samples infected with *F. graminearum* in which steeping water was changed displayed higher concentrations of (myco)toxins, especially ZEN and its derivates. This might be attributed to the inflow of essential minerals from the fresh water or arsenic as oxidative stress enhancer, but deeper, genetic studies should be employed in order to prove (or dismiss) this presumption. Additionally, an important finding is that even lower *F. graminearum* infection can result in heightened concentrations of some of the emerging mycotoxins (rubellin D, rugulusovin).

Malting and brewing processes have not been included into mycotoxins legislative per se, and this could represent a global health problem since beer is a widespread beverage. Multi-toxins can be found in malting and brewing by-products used as animal feed or additions to human nutrition [11,12]. Based on a few recent scientific papers published on mycotoxins in beer [10,11,12,13,19], an immediate update concerning the emerging multi-toxins that can be found in malt and beer, together with a risk assessment, is necessary.

## Figures and Tables

**Table 1:** General micromalting scheme of barley samples [4].

Day	Micromalting Step and Operating Conditions		Steeping Regime	
1	Immersion steeping for 5 h at 14 °C;		water change for batches A	no water change for batches B
	Dry steeping for 19 h at 14 °C, relative air humidity 95%.			
2	Immersion steeping for 4 h at 14 °C;		water change for batches A	no water change for batches B
	Dry steeping for 20 h at 14 °C, relative air humidity 95%.			
3	Immersion steeping for 1 h at 14 °C, relative air humidity 95%.		water change for batches A	no water change for batches B
3–6	Germination was carried out according to the scheme: 96 h at 14 °C Relative air humidity in each step was 95%			
7	Kilning was performed for 19 h, according to standard procedures for pale malt, after last germination hour;	50 °C for 16 h		
		60 °C for 1 h		
		70 °C for 1 h		
		80 °C for 1 h		
	Malt degermination; packing in paper bags and storage			

**Table 2:** The results of microbiological analysis of starting barley, finished malt, and mixed malt.

Starting Barley	Predicted Contamination Level, %	Actual Contamination Level, %
	0	0
Finished Malt		
AI	100	99
BI	100	100
AH	0	0
BH	0	0
Mixed Malt		
Steeping Water Change		
0	0	0
10	10	9
20	20	21
No Steeping Water Change		
0	0	0
10	10	11
20	20	22

AI = artificial infection with water change; BI = artificial infection withouth watre change; AH = no infection with water change; BH = no infection withouth water change.

**Table 3:** Concentrations of DON (deoxynivalenol) and its derivates (deoxynivalenol-3-glucoside and 3-acetyldeoxynivalenol) in malt samples.

Batch	Toxin (µg·kg^−1^)		
	DON	DON-3-GLC	3-ADON
Steeping Water Change			
0	25.4 ^f^	<LOD *	8.03 ^e^
10	282 ^d^	354 ^d^	14.4 ^d^
20	1001 ^a^	695 ^a^	110 ^a^
No Steeping Water Change			
0	38.1 ^e^	<LOD	5.10 ^f^
10	370 ^c^	407 ^c^	24.1 ^c^
20	685 ^b^	639 ^b^	84.5 ^b^

Values are means of triplicate. Values in the same column with different superscript letters (a–f) are significantly different (*p* < 0.05). * Limit of detection (LOD) for DON = 0.3 µg·kg^−1^; DON-3-GLC = 0.02 µg·kg^−1^; 3-ADON = 0.3 µg·kg^−1^.

**Table 4:** Concentrations of ZEN (zearalenone) and its derivatives (zearalenone-4-sulphate) in malt samples.

Batch	Toxin (µg·kg^−1^)			
	ZEN	ZEN-4-SULF	α-Zearalenol	β-Zearalenol
Steeping Water Change				
0	<LOD *	<LOD *	<LOD *	<LOD *
10	1252 ^b^	99.3 ^b^	9.23 ^c^	20.9 ^d^
20	2159 ^a^	1449 ^a^	25.1 ^a^	99.8 ^a^
No Steeping Water Change				
0	<LOD *	<LOD *	<LOD *	<LOD *
10	314 ^d^	19.3 ^d^	3.95 ^d^	28.0 ^c^
20	675 ^c^	48.5 ^c^	10.4 ^b^	41.0 ^b^

Values are means of triplicate. Values in the same column with different superscript letters (a–d) are significantly different (*p* < 0.05). * LOD values for ZEN = 0.03 µg·kg^−1^; ZEN-4-SULF = 1.6 µg·kg^−1^; α-zearalenol = 0.8 µg·kg^−1^; β-zearalenol = 1.2 µg·kg^−1^.

**Table 5:** Concentrations of other positively-identified mycotoxins/fungal metabolites in malt samples.

Toxin (µg·kg^−1^)			Steeping Water Change			No Steeping Water Change		
Batch	LOD µg·kg^−1^	LOQ * µg·kg^−1^	0	10	20	0	10	20
Abscisic acid	1.6	5.28	25.8 ^c^	36.3 ^a^	12.9 ^f^	20.7 ^d^	27.9 ^b^	18.7 ^e^
Alternariol	0.03	0.01	0.83 ^e^	5.85 ^d^	13.9 ^b^	<LOD	6.59 ^c^	16.7 ^a^
Alternariol methyl ether	0.01	0.03	<LOD	0.79 ^c^	1.78 ^b^	<LOD	0.65 ^d^	2.84 ^a^
Altersetin	0.4	1.32	10.7 ^e^	843 ^a^	573 ^b^	4.40 ^f^	253 ^d^	556 ^c^
Asterric acid	3.2	10.6	<LOD	<LOD	55.2 ^a^	<LOD	<LOD	<LOQ
Butenolide	5.6	18.5	<LOD	23.2 ^b^	25.8 ^a^	<LOD	<LOD	<LOQ
Brevianamid F	0.05	0.17	32.4 ^d^	33.9 ^b^	32.3 ^d^	32.6 ^c^	31.4 ^e^	34.1 ^a^
Chlamydosporol	0.5	1.65	1.65 ^e^	17.3 ^c^	22.1 ^a^	<LOD	7.39 ^d^	17.8 ^b^
Chlamydospordiol	0.16	0.53	<LOQ	<LOQ	2.58 ^b^	<LOQ	<LOQ	3.50 ^a^
Chlorocitreorosein	2	6.60	6.65 ^f^	15.1 ^c^	23.4 ^b^	11.1 ^e^	13.9 ^d^	24.2 ^a^
Citreorosein	0.64	2.11	2234 ^e^	2584 ^d^	3241 ^a^	1932 ^f^	2720 ^c^	3117 ^b^
Cordycepin	2	6.60	16.8 ^f^	18.0 ^e^	23.8 ^b^	19.4 ^d^	21.0 ^c^	24.1 ^a^
Dihydroxymellein	1.4	4.62	5.22 ^c^	5.59 ^b, c^	9.09 ^a^	4.65 ^d^	6.24 ^b^	9.45 ^a^
Emodin	0.005	0.02	<LOQ	6.15 ^d^	40.3 ^a^	<LOD	6.96 ^c^	25.5 ^b^
Epiequisetin	0.24	0.80	<LOD	<LOQ	1.74 ^a^	<LOD	<LOD	1.02 ^b^
Equisetin	0.24	0.80	36.8 ^c^	46.6 ^a^	41.8 ^b^	36.2 ^d^	42.3 ^b^	32.4 ^e^
Fellutanine A	0.64	2.11	<LOD	<LOD	230 ^a^	<LOD	33.5 ^c^	116 ^b^
Fusarin C	4.8	15.8	57.8 ^b^	96.0 ^a^	34.0 ^f^	45.2 ^e^	53.9 ^c^	46.1 ^d^
Infectopyron	6	19.8	24.6 ^d^	78.6 ^c^	176 ^a^	21.0 ^e^	20.6 ^e^	172 ^b^
Kojic acid	0.5	1.65	<LOD	10.0 ^c^	15.8 ^b^	<LOD	<LOQ	30.3 ^a^
Lotaustralin	2	6.60	7.82 ^d^	9.51 ^c^	10.0 ^b^	6.99 ^e^	9.90 ^b^	10.7 ^a^
Moniliformin	0.002	0.007	<LOD	<LOD	8.09 ^a^	<LOD	<LOD	<LOD
Nivalenol	0.03	0.10	9.04 ^f^	35.9 ^c^	61.5 ^a^	10.2 ^e^	21.6 ^d^	61.0 ^b^
Rubellin D	0.56	1.85	38.8 ^b^	45.1 ^a^	30.2 ^e^	37.3 ^c^	33.7 ^d^	34.0 ^d^
Rugulusovin	0.24	0.79	520 ^c^	11962 ^a^	665 ^b^	403 ^f^	508 ^d^	498 ^e^
Siccanol	0.4	1.32	<LOQ	950 ^a^	91.6 ^c^	<LOQ	343 ^b^	67.8 ^d^
Tenuazonic acid	3	10.0	91.2 ^c^	105 ^a^	77.5 ^e^	93.3 ^b^	83.4 ^d^	91.7 ^c^
Tryptophol	0.24	0.80	25.8 ^c^	36.3 ^a^	12.9 ^f^	20.7 ^d^	27.9 ^b^	18.7 ^e^

Values are means of triplicate. Values in the same column with different superscript letters (a–f) are significantly different (*p* < 0.05). * Limit of quantification (LOQ).

**Table 6:** General overview of detected toxins and their producers.

Producer		Mycotoxin
*Fusarium* spp.		3-acetyl-deoxynivalenol
		Butenolide
		Chlamydosporol
		Chlamydospordiol
		Deoxynivalenol
		Epiequisetin
		Equisetin
		Fusarin C
		Moniliformin
		Nivalenol
		Siccanol
		α-zearalenol
		β-zearalenol
		Zearalenone
*Alternaria* spp.		Alternariol
		Alternariol methyl ether
		Altersetin
		Infectopyron
		Tenuazonic acid
Unspecific		Brevianamide F
		Citreorosein
		Emodin
		Rugulusovin
		Chlorocitreorosein
		Tryptophol
		Fellutanine A
Plant		Abscisic acid
		Lotaustralin
	Modified	Deoxynivalenol-3-glucoside
		Zearalenone-4-sulphate
Other fungal species		Asterric acid
		Dihydroxymellein
		Kojic acid
		Rubellin D
